# Strangulated Hernia

**Published:** 1871-09

**Authors:** W. F. Westmoreland

**Affiliations:** Professor of Surgery in Atlanta Medical College


### STRANGULATED HERNIA.

*Removal of Three and a Half Ounces of Omentum.*

By W. F. Westmoreland, M.D.,
Professor of Surgery in Atlanta Medical College.

On Tuesday, the 28th of June, I was requested by Dr. W. N. Judson, of this city, to see with him Mr. B. K——, a stout, muscular man, twenty-six years old. Upon my arrival, at five o'clock A.M., I learned from Dr. Judson that Mr. K. was attacked, forty hours previously, by what he (the patient) supposed colic. Various domestic remedies had been taken, without affording relief. The patient continued to grow worse up to twelve o'clock Monday night, at which time Dr. Judson was called, and found him suffering intense pain over the entire abdomen, and vomiting occasionally. Upon examination, a tumor half the size of a fœtal head, and partially descending into the scrotum, was found in left inguinal region. The pain and distress continued to increase, and the ejections from the stomach, after a short time, were accompanied by a peculiar fæcal odor, that showed conclusively the character of vomiting. I found in Mr. K. all the usual symptoms, well-marked, of strictured bowel. I learned, further, that for fifteen years he had been troubled by oblique inguinal hernia of left side; that for the last three years the hernia had been irreducible, and that the tumor, which had never descended into the scrotum, had greatly increased in size since the present attack.

After an unsuccessful effort at taxis, we ordered one grain of sulphate of morphine to be given at once, and cold to be constantly applied to hernial tumor by means of a bladder partially filled with pounded ice. In two hours we returned, and found the tumor considerably reduced, and the patient decidedly more comfortable. Another prolonged and unsuccessful effort to reduce the bowel was made. We ordered another grain of morphine, and a continuation of the ice application. In three hours we returned, found the tumor about the size it was when we last saw it, and the patient semi-narcotized. After another unsuccessful effort at taxis, all hope of reduction by this mode was abandoned.

An operation was proposed, and readily accepted by the friends of the patient. At two P.M. of the same day, assisted by Drs. Judson and D'Alvigny, I opened the sac, bringing to view a mass of omentum. While carefully unrolling its folds, a knuckle of bowel (three or four inches) of dark mahogany hue was found, which readily returned after the strictured ring had been divided in the usual way.

The omentum was considerably congested, and at some points the circulation completely arrested. We removed, with scissors, such parts as we feared would slough, and attempted to return the mass. In this we failed, even after an extension of the incision of the strictured neck of the sack. A more careful examination revealed extensive adhesions of the omentum and neck of the sac. It was at once determined to remove the entire mass. As vessels of considerable size supplied the extended omentum, it was ligatured with silk cord, as near the internal ring as possible, before the knife was used. The portion removed, after being disgorged of much blood by the division of vessels, weighed three ounces and a half. The stump receded to internl ring, and the ligature, of sufficient length to pass through the external wound, was secured by adhesive strip. A pledget of lint was placed in the wound, for the purpose of securing proper drainage. In one hour, the patient had a free action of the bowels. An anodyne was administered.

Early next morning I was called, and learned that the patient had been greatly annoyed by frequent discharges from bowels of fœcal matter, mucous and bloody serum. For four days, notwithstanding all our efforts, the discharges increased in frequency. On the third and fourth days, they were of hourly occurrence. At night of the fourth day, he was so much exhausted that it seemed impossible for him to recover. Fortunately, however, the diarrhea—or, rather, the inflammation of the mucous membrane of the bowel—gradually subsided and, at the end of the first week, disappeared.

Was this inflammation the result of mechanical injury to the bowel by the strictured ring, or was it produced by the cathartics taken, without medical advice, during the first twenty-four hours of his trouble, or both? At no time were there symptoms of peritonitis.

Three weeks after the operation, a small abscess formed near the ligatured stump, which was opened just over the internal ring. The ligature came way on the thirty-fifth day. It is now two months since the operation. The patient has entirely recovered.

